# Rasch models to assess the impact of lack of measurement invariance and reveal hidden differences in anxiety and depression between groups and over time in patients with early-stage melanoma or breast cancer using the RespOnse Shift ALgorithm at the Item level (ROSALI)

**DOI:** 10.1186/s12874-025-02756-2

**Published:** 2026-01-22

**Authors:** Yseulys Dubuy, Myriam Blanchin, Bastien Perrot, Marianne Bourdon, Véronique Sébille

**Affiliations:** 1 Nantes Université, Université de Tours, INSERM, MethodS in Patients-centered outcomes and HEalth Research, SPHERE, Nantes, F-44000 France; 2 CHU Nantes, DRCI, Methodology and Biostatistics Department, Nantes, France; 3 Institut de Cancérologie de l’Ouest (ICO), Nantes, Angers, France

**Keywords:** Breast cancer, Melanoma, Anxiety, Depression, Differential item functioning, Response shift

## Abstract

**Background:**

Patient-reported outcome measures (PROMs) are often challenging to analyze and interpret. Indeed, patients may give different answers to PROMs over time, not only because their level of the target construct (e.g. anxiety) has changed but also because their interpretation of the items aiming at measuring the construct has changed. For instance, cancer treatment may trigger changes in the patients’ internal standard of measurement (i.e., recalibration response shift, RS), resulting in a lack of measurement invariance over time. In addition, interpretation of PROMs items may differ according to cancer type (differential item functioning, DIF). If ignored, DIF and RS may impact inferences made from PROMs; they are also crucial to investigate as they may be related to patients’ adaptation after a salient health event, e.g. cancer diagnosis. Our objectives were to show how cross-sectional and longitudinal Rasch models can be used to detect, interpret, and account for DIF and RS, where appropriate, when measuring anxiety and depression in breast cancer and melanoma patients.

**Methods:**

Anxiety and depression were assessed in breast cancer (*n* = 337) and melanoma patients (*n* = 110) using the Hospital Anxiety and Depression Scale (HADS) at 1- (T1) and 6-month (T2) post-diagnosis. DIF and RS analyses were performed using the RespOnse Shift ALgorithm at the Item level (ROSALI) based on Rasch models, i.e. cross-sectional and longitudinal Partial Credit Models (PCM).

**Results:**

DIF and RS were identified in the anxiety (DIF and RS) and depression (RS only) subscales of the HADS. DIF and RS had a moderate (anxiety) or significant impact (depression) on the results, providing different conclusions depending on whether or not they were considered. More specifically, wrongly presuming (longitudinal) measurement invariance would have resulted in overestimating the increase in depression over time among breast cancer patients.

**Conclusion:**

The ROSALI procedure based on Rasch models is freely available (https://pro-online.net/). ROSALI enabled to investigate measurement invariance at item-level, providing insight into cancer patients’ experience, possibly revealing psychological distress but also psychological adaptation to challenging health events. Although some methods are available, DIF and RS are still too often ignored which can lead biased measurements of constructs and to suboptimal healthcare decision making.

**Trial registration number:**

n°CT.gov: NCT02893774, registration date: 2016-08-25. Of note, this was a prospective study which was retrospectively registered.

**Supplementary Information:**

The online version contains supplementary material available at 10.1186/s12874-025-02756-2.

## Introduction

Patient-Reported Outcome Measures (PROM), such as questionnaires composed of items regrouped in different dimensions, are directly completed by patients to better understand their subjective experience of illness and of its healthcare management. PRO (e.g. health-related quality of life (HRQoL), anxiety, depression) are considered essential to patient-centered care to assess important aspects of patients’ lives and offer a holistic approach to patient care [1, 2].

Yet, to contribute to improving patient-centered care, PROM must measure what they are supposed to measure and measure the same thing over time, regardless of patients’ characteristics. However, patient responses to PROM are sometimes not consistent with what was expected (e.g. a good reported HRQoL despite a deteriorated health state). One possible explanation is that patients with different clinical characteristics or life stories may not understand/interpret questions about their experience in the same way at a given time (i.e. differential item functioning, DIF) [3]. In addition, their interpretation may also change over time (i.e. response shift, RS) [4, 5]. Indeed, people exposed to distressing events such as cancer diagnosis and treatments may reappraise the meaning of important aspects of their lives and this may result in DIF or RS in the measurement of the target constructs of interest, e.g. anxiety or depression. Hence, in case of RS (DIF), the observed changes over time (differences between groups) in items responses or dimension scores may not fully reflect changes (differences) in the target constructs [5].

While DIF or RS could either be a result of positive adaptation or reveal adjustment disorders to health challenges that are worth detecting, these phenomena, if ignored, can also lead to biased interpretations about self-reported outcomes and to suboptimal healthcare decision-making [6–8]. Ignoring RS or DIF may indeed have two implications. First, it may result in biased estimations of changes/differences in the target construct of interest and therefore to erroneous conclusions. Second, patients’ adaptation to their disease and healthcare would then be overlooked. In either case, if RS and/or DIF are ignored, information regarding experiences that may be meaningful to patients and healthcare providers may be lost [7–9].

A broad range of methods exist for detecting DIF in the literature. As most of these methods are older and generally better known than those dedicated to RS detection, the interested reader can turn to the enlightening overviews provided by Millsap [10], French & Finch [11], and Jones [12]. Several quantitative methods have been proposed to detect RS such as design-based methods [13, 14] (which needs to be planned in advance as they require specific materials, e.g. specific questionnaires), latent variable models [15, 16], or regression-based models [17–19]. Some methods only focus on RS detection, while some others also allow for estimating the magnitude of RS effects and taking RS into account in the analyses. Among these methods, the most prevalent are the then-test [20] and structural equation models (SEM) [15], which are a design-based and a latent variable methods, respectively [21, 22]. SEM is a more versatile method than the then-test as it can be used in studies where RS is the main aim [23, 24] but also in secondary analyses, for instance, to investigate whether RS could have had an impact on the interpretation of initial findings [25, 26]. Moreover, SEM can, in principle, operationalize the three types of RS, i.e. recalibration (a change in the patient’s internal standards of measurement), reprioritization (a change in the importance of dimensions/items constituting the target construct of interest, e.g. anxiety), and reconceptualization (a redefinition of the target construct) [4]. Although SEM are often used to perform dimension-level analyses, they can also be adapted to conduct item-level analyses, offering a greater granularity [27, 28].

A more recent latent variable method for RS at the item level, named the RespOnse Shift ALgorithm at the Item level (ROSALI), based on either Item Response Theory (IRT) [16] or Rasch Measurement Theory (RMT) [29], was developed and its performance for RS detection at the item level was evaluated using simulation studies mimicking health research studies including PROM [29, 30]. ROSALI allows for detecting and accounting for recalibration RS in the analyses, where appropriate. One of these simulation studies was specifically dedicated to the comparison of ROSALI, either based on IRT or RMT, and SEM [29]. This study showed that ROSALI, based on RMT, had better performance than ROSALI based on IRT and SEM with regards to: (i) recalibration RS detection and (ii) falsely concluding that RS had occurred. ROSALI based on RMT has subsequently been extended to include one binary covariate in order to investigate whether RS occurs differently or similarly in two groups [31]. This extension was shown to have good performance for RS detection and provided unbiased estimations of the change in the target construct, as assessed within another simulation study [30].

There are some theoretical arguments and empirical indications of DIF and RS in cancer patients [31–33]. For instance, DIF by cancer type and gender was found when assessing coping strategies among cancer survivors [32] using cross-sectional RMT models. In this study, patients seemed to perceive and interpret some items differently depending on these characteristics. Henceforth, the responses provided by patients to the items may not only have reflected their coping strategies but also different expectations or meanings related to coping, by cancer type and gender. Similarly, RS has been reported in studies including patients with, e.g., prostate [24], breast [34], or lung [35] cancer, or in studies mixing different cancer types [31, 33, 36] using mostly longitudinal SEM, ROSALI based on RMT or the then-test. For instance, Hammas et al. [31] used ROSALI based on RMT and highlighted DIF and RS when assessing HRQoL by cancer type (breast cancer and melanoma). Specifically, the authors showed that change in item responses reflected not only a change in emotional functioning, but also changes in item interpretation, over time, and between cancer types.

As a life-threatening disease, cancer is known to have profound psychological and psychiatric implications which may in turn influence HRQoL, treatment adherence, and survival [37, 38]. Anxiety and depression are reported as being the most frequent psychiatric comorbidities in cancer patients [39–41]. Even if the factors associated with depression and anxiety in cancer still remain to be characterized, cancer type seems to be essential to consider [41]. Suggested causes for the influence of cancer type on anxiety or depression include the different degrees of body image disruption, differing prognosis, or tumor-related neuropsychiatric side effects [39]. The present study focuses on two types of cancer (breast cancer and melanoma) that have a good prognosis in the early stages, but whose treatments and symbolism carried by the affected organ are different [42]. We hypothesized that DIF and RS could occur and contribute to the observed differences in anxiety and depression according to cancer type, particularly before and during the treatment phase.

Our first objective was to identify and quantify DIF and RS using ROSALI based on RMT in self-reported measures of anxious and depressive symptoms in breast cancer and melanoma patients in an acute phase (treatment course) of the disease where these symptoms were reported as prevailing [43]. Our second objective was to assess the impact of DIF and RS on the estimates of differences and changes in anxiety and depression in breast cancer and melanoma patients.

## Materials and methods

### Participants

The ELCCA study (NCT02893774) aims at examining socioeconomic, psychological, and HRQoL changes following diagnosis of breast cancer or melanoma (stages I, II: early stage, non-metastatic) [44]. The study took place in France (Nantes Integrative Center for Oncology, for breast cancer patients and Department of Onco-dermatology at Nantes University Hospital, for melanoma patients) and was approved by a local ethical research committee (“Comité de Protection des Personnes”). Informed consent was obtained from all patients before inclusion; inclusions occurred between 2010 and 2016.

Patients’ follow-up was initially planned to last until two years post-cancer diagnosis, with a follow-up at 1, 6, 12 and 24 months. Follow-up has then been extended for some patients, to explore long term post-diagnosis changes (up to 10 years post-diagnosis). Our study focuses on an acute phase of the disease, i.e. during treatment course (from 1- to 6-month post-diagnosis).

As melanoma and breast cancer were at an early stage, the prognosis was considered good for both. However, patients had different treatments: potentially invasive for breast cancer (often involving major surgery and radio/chemo/hormonal therapies) and less invasive for melanoma (minor surgery possibly followed by immunotherapy) [44].

### Measures

Participants completed self-reported questionnaires, among which the Hospital Anxiety and Depression scale (HADS) [45] over the course of their follow up. The HADS is a self-reported questionnaire measuring anxiety and depressive symptoms. It contains 14 items divided into two subscales: 7 items in the anxiety (HADS-A) and depression (HADS-D) subscales, respectively. Each item is scored on a 4-point rating scale ranging from 0 to 3. Patients are asked to respond on the basis of how they have been feeling in the past week. Response categories 0 and 3 reflect a low and high level of anxiety or depression, respectively.

### Statistical analysis

#### Sample description

Continuous data were summarized by means and standard deviations. Categorical data were expressed by frequencies and percentages. Characteristics of melanoma and breast cancer patients were compared using t-tests and chi-square tests for continuous and categorical data, respectively.

#### DIF and RS detection for anxiety and depression

DIF and RS are special cases of lack of measurement invariance [5] which occurs when the distribution of the responses to an item not only depends, as expected, on the level of the target construct of interest (e.g. anxiety), but is also influenced by other variables, such as cancer type for DIF (i.e. between-group lack of measurement invariance), or time of measurement for RS (i.e. lack of longitudinal measurement invariance).

We used ROSALI based on RMT [31] (hereafter referred to as the ROSALI procedure) to investigate DIF and recalibration RS among the items of the HADS-A and HADS-D subscales in melanoma and breast cancer patients and estimate difference and change over time in the target construct while adjusting for DIF and RS, if appropriate. Analyses were performed separately for both subscales of the HADS. The occurrence of DIF and recalibration RS was evaluated during the treatment period: between 1- and 6-month post-diagnosis (denoted T1 and T2, respectively), in the two groups formed by melanoma (G1) and breast cancer patients (G0, reference group in our analysis).

#### Overview of the ROSALI procedure

**Fig. 1:**
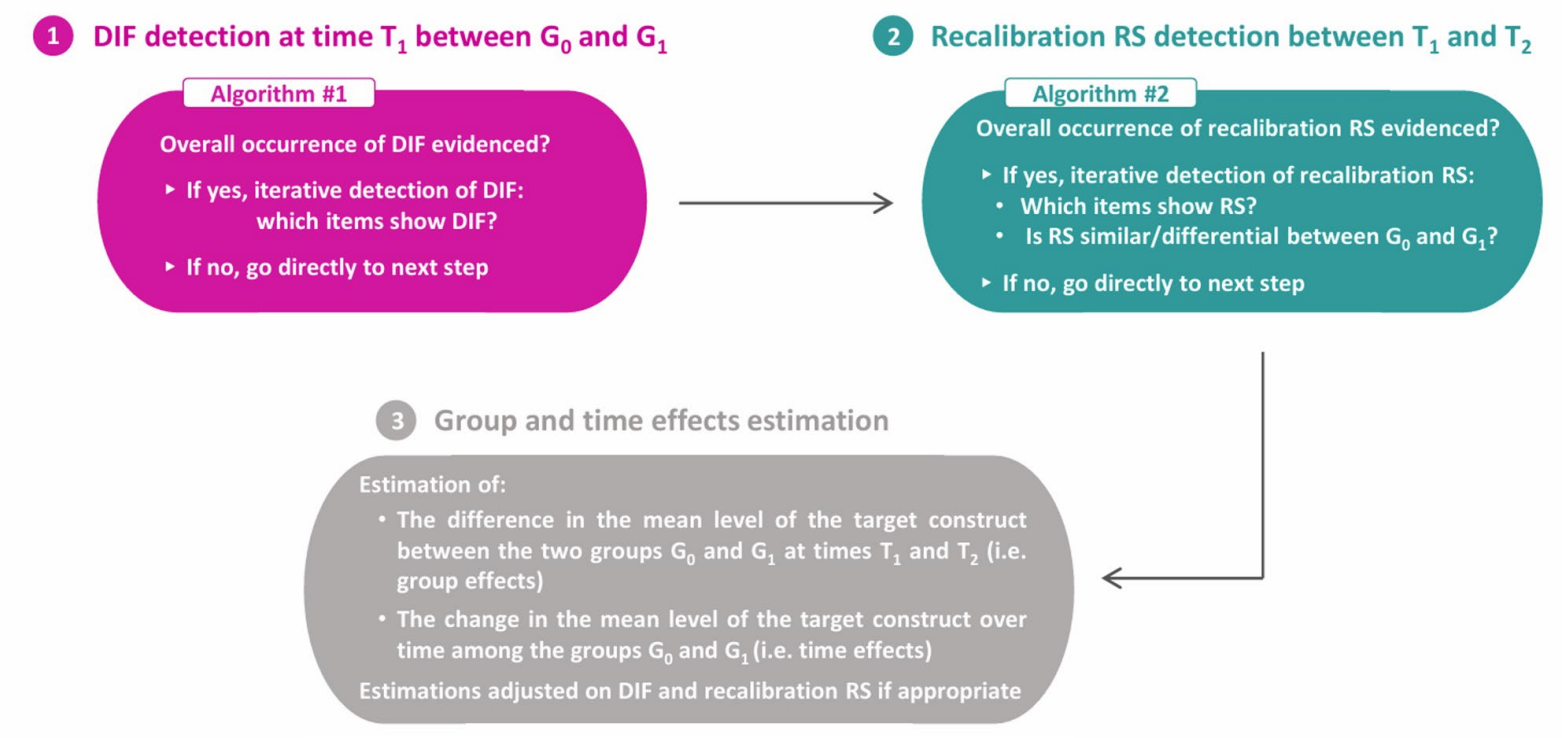
The ROSALI procedure with one binary covariate G and two time points T1 and T2. Within our manuscript, covariate G = Cancer type (G0 = Breast cancer patients and G1 = Melanoma patients), time T1 and T2 = one and six months after cancer diagnosis, respectively. *Adapted from Hammas et al. Frontiers in Psychology*,* 2020*

The ROSALI procedure (depicted in Fig. 1) is an iterative item-by-item procedure that comprises two sequential algorithms which are dedicated to the detection of:

1. DIF between the two groups G0 and G1 at T1 (algorithm #1, first part of the ROSALI procedure).
2. Recalibration RS between T1 and T2 in each group (algorithm #2, second part of the ROSALI procedure). In case of recalibration RS, it is possible to assess whether recalibration is similar in both groups or occurs differently (e.g. occurs only in one group or differently in the two groups).

Once DIF and recalibration RS detection is performed, ROSALI can provide estimates of:

- The difference in the mean level of anxiety and depression between the two groups at T1 and T2 (i.e. group effects).
- The change over time in the mean level of anxiety and depression among the groups (i.e. time effects).

All these estimations are adjusted for DIF and RS, if appropriate.

#### Models involved in the ROSALI procedure

ROSALI is based on the estimation of several Partial Credit Models (PCM), which are probabilistic models from the RMT dedicated to polytomous items [46, 47]. Taking the anxiety subscale of the HADS as an illustration and without loss of generality, patient responses to the HADS-A subscale are modelled as a function of a continuous variable $\:{\Theta\:}$, called ‘latent variable’ assumed to represent the level of anxiety (i.e. the target construct of interest). Specifically, it is assumed that the higher the level of the latent variable, the higher the individual’s response to an item. The PCM formulation is given below for a set of $\:J\:$polytomous items (item1, ., item $\:J)$, each having $\:{M}_{j}+1$ response categories (0,1, …, $\:{M}_{j})$:

1 $$P\left({X}_{ij}=x|{\theta}_{i},\:{\delta}_{j1},\dots\:,\:{\delta}_{j{M}_{j}}\right)=\frac{exp(x{\theta}_{i}-\sum_{p=1}^{x}{\delta}_{jp})}{\sum_{l=0}^{{M}_{j}}exp(l{\theta}_{i}-\sum_{p=1}^{l}{\delta}_{jp})}$$

where $\:{X}_{ij}$ is the response of individual $\:i\:$to item $\:j$, $\:{\theta}_{i}$ denotes his/her/their latent variable level (realization of $\:{\Theta\:}$), and $\:{\delta}_{jp}$ ($\:p=$1,…,$\:{M}_{j})$ are the threshold parameters associated with item $\:j$. Each item is characterized by $\:{M}_{j}$ threshold parameters and for a given $\:p\:$in $\:\{1,\dots\:,{M}_{j}\}$, $\:{\delta}_{jp}$ represents the latent variable level at which the probabilities of selecting category $\:p$ or category $\:p-1$ for item $\:j$ are equal. Of note, the latent variable $\:{\Theta\:}\:$is assumed to be normally distributed (with a mean $\:\mu\:=0$ for identifiability constraint with the marginal maximum likelihood estimation). 

**Fig. 2:**
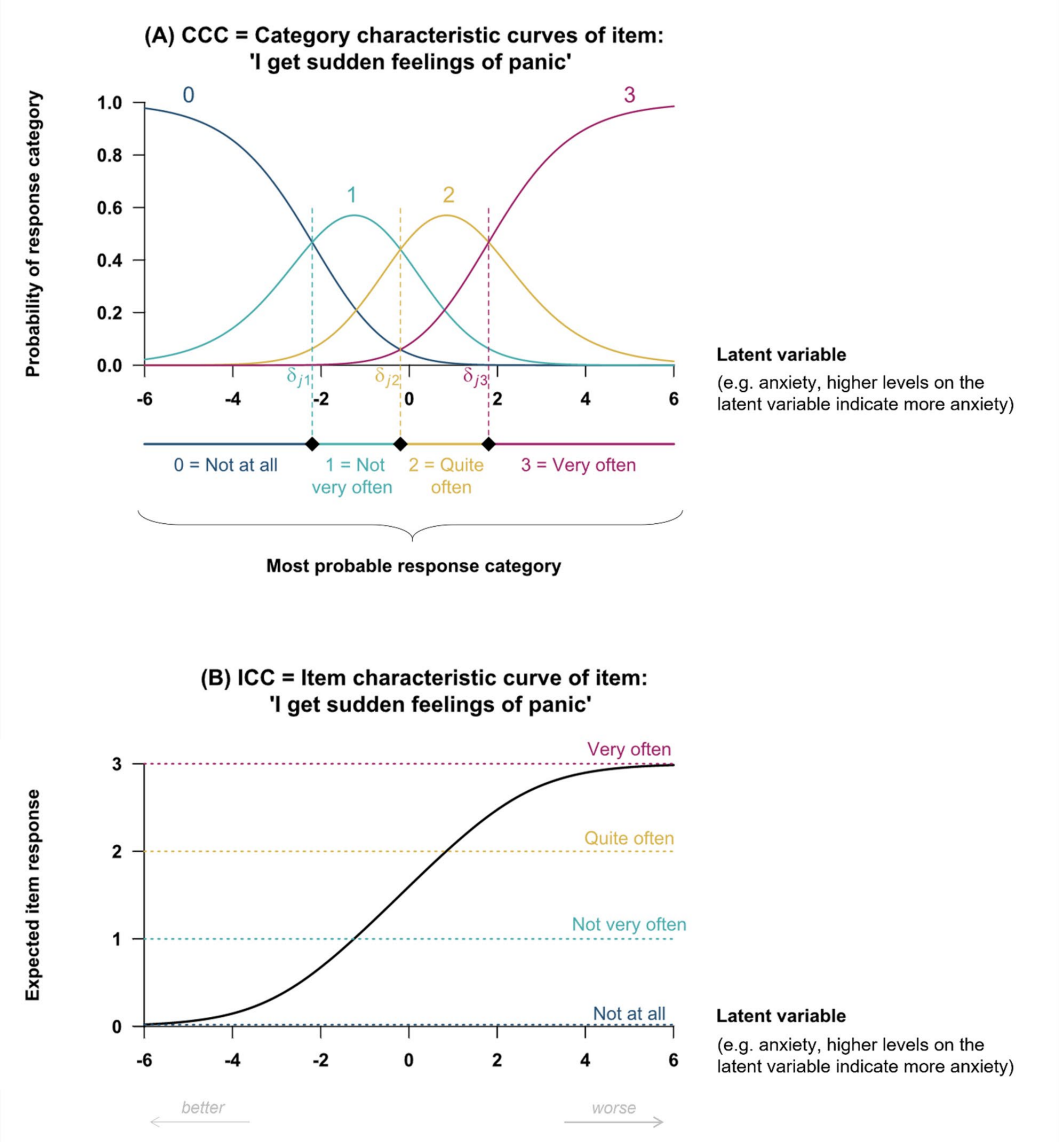
Category characteristic curves (graph **A**) and item characteristic curve (graph **B**) of an item targeting anxiety (e.g. item = ‘*I get sudden feelings of panic*’) with 4 ordered response categories (0 = ‘*Not at all*’, 1 = ‘*Not very often*’, 2 = ‘*Quite often*’, 3 = ‘*Very often*’) under the Partial Credit Model (PCM). Vertical dashed lines in Graph (**A**) represent the threshold parameters $\:{\delta}_{jp}$, characterizing the item (i.e. the latent variable level at which two adjacent category characteristic curves intersect)

Two possible graphical representations of the PCM, named respectively the *category characteristic curves* (CCC) and the *item characteristic curve (ICC)*, are provided in Fig. 2A (CCC) and 2B (ICC), for an item $\:j$ with $\:{M}_{j}+1=\:$4 ordered response categories (0, 1, 2, 3) and hence $\:{M}_{j}=\:$3 threshold parameters (e.g. the HADS-A item ‘*I get sudden feelings of panic*’: 0 = ‘*Not at all*’, 1 = ‘*Not very often*’, 2 = ‘*Quite often*’, 3 = ‘*Very often*’ targeting anxiety). Figure 2A represents the probability of selecting each response category of item j according to the level of the latent variable (e.g., the level of anxiety). There are four category characteristic curves: one for each response category (0,1, 2, 3). Based on the PCM, the lowest response category 0 = *‘Not at all*’ is more likely to be selected for low level of anxiety (e.g., when the latent variable is about $\:-\mathrm{4}$). Conversely, the highest response category 3 = ‘*Very often*’ is more likely to be selected for high level of anxiety (e.g., when the latent variable is about $\:\mathrm{4}$). The three threshold parameters are highlighted by the vertical dashed lines. Figure 2B provides the expected response to the item for each latent variable level. The expected item response (computed as the mathematical expectation of $\:{X}_{ij})$ increases with increasing latent variable level.

Within the ROSALI procedure considering a binary group covariate G and two time points T1 and T2, two different types of PCM are estimated:

- **Cross-sectional PCM at time T1, allowing to detect DIF between G0 and G1 at time T1 (algorithm #1):**
  These models are based on cross-sectional PCM including the effect of covariate G on the latent variable level, as in the formula hereafter in case of group-invariance (no DIF):
  2 $$P\left({X}_{ijg}^{}=x|{\theta}_{{g}_{i}}^{},{\delta}_{j1},\dots\:,\:{\delta}_{j{M}_{j}}\right)=\frac{{exp}\left(x{\theta}_{{g}_{i}}-\sum_{p=1}^{x}{\delta}_{jp}\right)}{\sum_{l=0}^{{M}_{j}}{exp}\left(l{\theta}_{{g}_{i}}-\sum\:_{p=1}^{l}{\delta}_{jp}\right)}$$
  Where:
  $\:g$ is either equal to 0 or 1 (the two groups: 0 = breast cancer [reference], 1 = melanoma)
  $\:{X}_{ijg}^{}$ denotes the response of individual $\:i\:$to item $\:j$ in group $\:g$.
  $\:{\theta}_{{g}_{i}}^{}\:$is the latent variable level of individual $\:i\:$in group $\:g$; it is a realization of the latent variable $\:{{\Theta}}_{g}^{}\:\sim\:\mathcal{N}({\mu}_{g},\:{\sigma}_{g}^{2})$ with:
  - $\:{\mu}_{0}$, the mean in group G0, is set to 0 (identifiability constraint for reference group).
  - $\:{\mu}_{1}$, the mean in group G1, is freely estimated.
  - $\:{\sigma}_{0}^{2}$ = $\:{\sigma}_{1}^{2}=\sigma^{2}$ (the ROSALI procedure assumes identical variances of the latent variable across groups).
  This cross-sectional PCM allows to account for the possibility that the group covariate G (cancer type) might explain some of the variation in the latent variable (e.g., anxiety, depression) through the estimation of group-specific means. The ‘*group effect’* of the covariate $\:G\:$on the latent variable at time T1 is therefore given by $\:{\mu}_{1}-\:{\mu}_{0}=\:{\mu}_{1}$. It corresponds to the group differences in the latent variable means.
  Based on this model, DIF can be operationalized and investigated at time T1 on each item $\:j$ by freely estimating its threshold parameters across the two groups (i.e. allowing all thresholds $\:{\delta}_{jp}\:$of the item under investigation to vary between the two groups: $\:{\delta}_{j{p}_{0\:}}$among G0 and $\:{\delta}_{j{p}_{1\:}}$among G1, $\:p\in\:\{1,\dots\:,{M}_{j}\}$):
  3 $$P\left({X}_{ijg}^{}=x|{\theta}_{{g}_{i}}^{},{{\delta}_{j1}}_{g},\dots\:,\:{{\delta}_{jM_j}}_{g}\right)=\frac{{exp}\left(x{\theta}_{{g}_{i}}-\sum_{p=1}^{x}{\delta}_{{jp}_{g}}\right)}{\sum_{l=0}^{{M}_{j}}{exp}\left(l{\theta}_{{g}_{i}}-\sum_{p=1}^{l}{\delta}_{{jp}_{g}}\right)}$$
  Of note, if DIF is assumed to occur on all items (i.e. all item thresholds are freely estimated), then $\:{\mu}_{1}$, and consequently the group effect on the latent variable level, cannot be estimated; it is therefore set to 0 (identifiability constraint).

- **Longitudinal PCM between T1 and T2, allowing to detect recalibration RS over time (algorithm #2):**
  These models are based on longitudinal PCM including the effects of covariate G and measurement occasion on the latent variable level, as in the formula hereafter in case of group and longitudinal invariance (no DIF, no RS):
  4 $$\:P\left({X}_{ijg}^{\left(t\right)}=x|{\theta}_{{g}_{i}}^{\left(t\right)},{\delta}_{j1},\dots\:,\:{\delta}_{j{M}_{j}}\right)=\frac{{exp}\left(x{\theta}_{{g}_{i}}^{\left(t\right)}-\sum_{p=1}^{x}{\delta}_{jp}\right)}{\sum_{l=0}^{{M}_{j}}{exp}\left(l{\theta}_{{g}_{i}}^{\left(t\right)}-\sum_{p=1}^{l}{\delta}_{jp}\right)}$$
  This model now enables to consider both time points T1 and T2 in addition to the binary covariate. $\:{X}_{ijg}^{\left(t\right)}\:$and $\:{\theta}_{{g}_{i}}^{\left(t\right)}$ denote, respectively, the response of individual $\:i$ in group $\:g\:$to item $\:j$ and his/her/their latent variable level at time $\:t$ (where $\:t=$ T1 or T2). $\:{\theta}_{{g}_{i}}^{\left(t\right)}$ is a realization of $\:{{\Theta}}_{g}^{\left(t\right)}$ with:
  $$\:\left(\begin{array}{c}{{\Theta}}_{g}^{\left(T1\right)}\\\:{{\Theta}}_{g}^{\left(T2\right)}\:\end{array}\right)\:\sim\:\mathcal{N}\left(\left(\begin{array}{c}{\mu}_{g}^{\left(T1\right)}\\\:{\mu}_{g}^{\left(T2\right)}\:\end{array}\right),\left(\begin{array}{cc}{{\sigma}_{g}^{2}}^{\left({T}_{1}\right)}&\:{{\sigma}_{g}^{}}^{\left({T}_{1},{T}_{2}\right)}\\\:{{\sigma}_{g}^{}}^{\left({T}_{1},{T}_{2}\right)}&\:{{\sigma}_{g}^{2}}^{\left({T}_{2}\right)}\end{array}\right)\right)$$
  In ROSALI, we have:
  - The mean in group G0 at T1 ($\:{\mu}_{0}^{\left(T1\right)}$) set to 0 (identifiability constraint).
  - All other latent means freely estimated.
  - The variances of the latent variable assumed equal between groups at each time point: $\:{{\sigma}_{0}^{2}}^{\left({T}_{1}\right)}={{\sigma}_{1}^{2}}^{\left({T}_{1}\right)}$ and $\:{{\sigma}_{0}^{2}}^{\left({T}_{2}\right)}={{\sigma}_{1}^{2}}^{\left({T}_{2}\right)}$.
  - The variances allowed to vary over time.
  - The covariance between both time points are assumed identical across G0 and G1: $\:{{\sigma}_{0}^{}}^{\left({T}_{1},{T}_{2}\right)}=\:{{\sigma}_{1}^{}}^{\left({T}_{1},{T}_{2}\right)}$
  At each time point $\:t,\:$the group effect on the latent variable level is given by $\:{\mu}_{1}^{\left(t\right)}-\:{\mu}_{0}^{\left(t\right)}$ (group difference in the latent means at this given time). Furthermore, time effect can be computed for each group, as the change in the latent variable mean over time:
  - $\:{\mu}_{0}^{\left(T2\right)}-\:{\mu}_{0}^{\left(T1\right)}=\:{\mu}_{0}^{\left(T2\right)}\:$in group G0 (cf. identifiability constraint: $\:{\mu}_{0}^{\left(T1\right)}=0$).
  - $$\:{\mu}_{1}^{\left(T2\right)}-\:{\mu}_{1}^{\left(T1\right)}\:$$in group G1.
  Based on this model, recalibration RS between T1 and T2 can be operationalized and investigated on each item $$\:j$$ and within each group (G0 and G1) by freely estimating its threshold parameters over time in both groups (i.e. allowing the thresholds $$\:{\delta}_{jp}\:$$of the item under investigation to vary between both time points among G0 and among G1; $$\:{\delta}_{{jp}_{g}}^{\left({T}_{1}\right)}\ne\:$$$$\:{\delta}_{{jp}_{g}}^{\left({T}_{2}\right)}$$):
  5 $$\:P\left({X}_{ijg}^{\left(t\right)}=x|{\theta}_{{g}_{i}}^{\left(t\right)},{\delta}_{j{1}_{g}}^{\left(t\right)},\dots\:,\:{\delta}_{j{M}_{{j}_{g}}}^{\left(t\right)}\right)=\frac{{exp}\left(x{\theta}_{{g}_{i}}^{\left(t\right)}-\sum_{p=1}^{x}{\delta}_{j{p}_{g}}^{\left(t\right)}\right)}{\sum_{l=0}^{{M}_{j}}{exp}\left(l{\theta}_{{g}_{i}}^{\left(t\right)}-\sum_{p=1}^{l}{\delta}_{j{p}_{g}}^{\left(t\right)}\right)}$$
  Recalibration RS is said to occur differently between G0 and G1 if the threshold parameters of the item under investigation change differently between the two groups. It is said to occur similarly otherwise. Of note, if recalibration RS is assumed to occur on all items in group G = G0 or G1 (i.e. the threshold parameters of all items are freely estimated over time in the group G), then $\:{\mu}_{g}^{\left(T2\right)}$, and consequently the time effect on the latent variable level among this group, cannot be estimated. It is therefore set to 0 (identifiability constraint).
  As mentioned above, ROSALI is an iterative procedure composed of two sequential algorithms. Consequently, each step in ROSALI takes into account the DIF/RS effects evidenced during the previous steps.

#### Graphical representation of DIF/RS effects

**Fig. 3:**
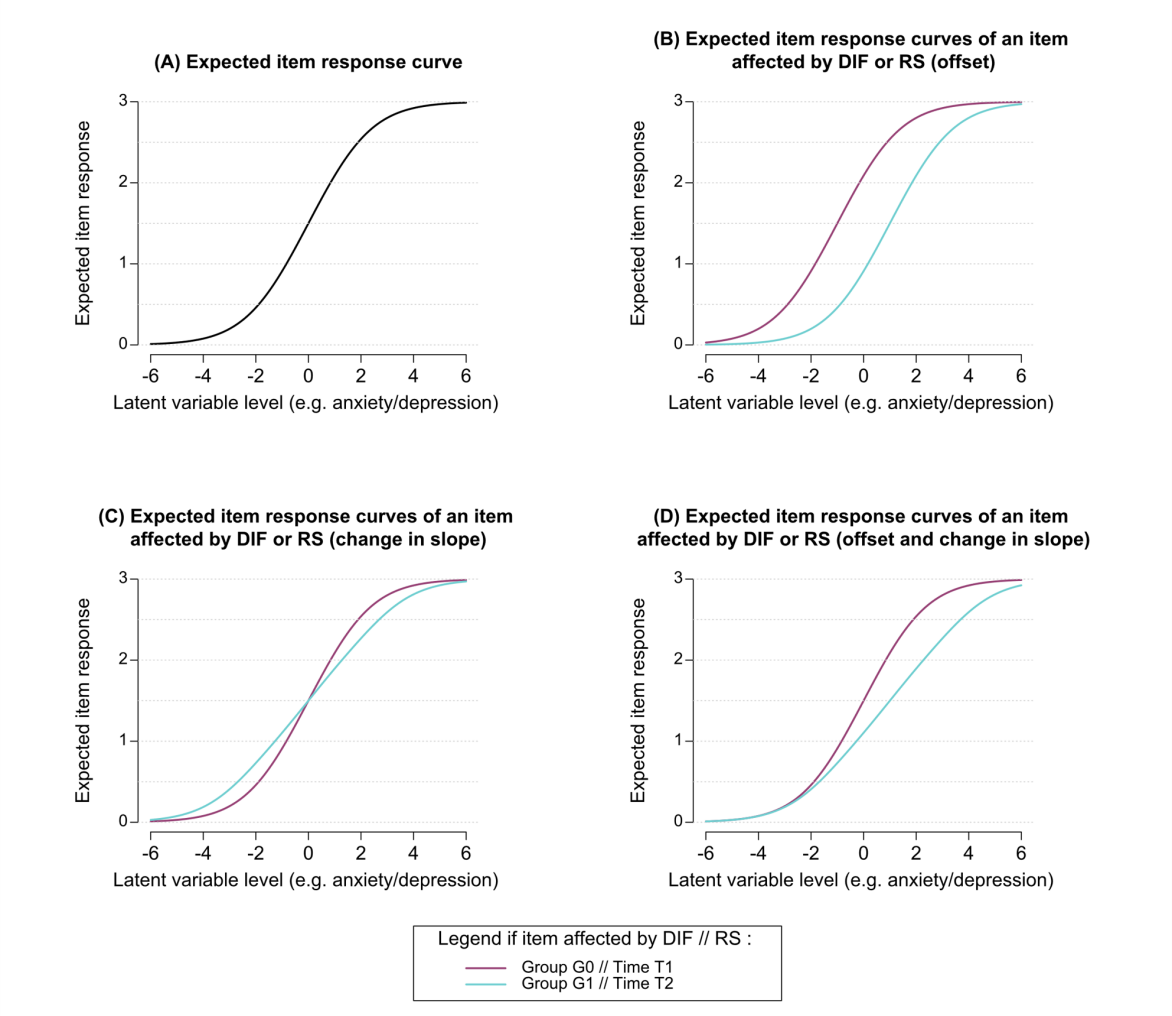
Item characteristic curves (i.e., expected item response curves) of an item with 4 response categories (0, 1, 2, 3) affected either by differential item functioning (DIF) related to the binary covariate G or recalibration response shift (RS) between time points T1 and T2. Graph (**A**): Expected item response curve assuming no differential item functioning (DIF) and no response shift (RS): the two curves overlap. Graphs (**B**), (**C**) and (**D**): expected item response curve of an item affected by DIF or RS: the two curves do not overlap, different patterns of the curves and item responses may be obtained despite having a same level of anxiety or depression between groups or over time (e.g. level of anxiety or depression equal to 2). Figure notes: the two curves of graphs (**B**), (**C**) and (**D**) represent either: the expected item response curves in groups G0 and G1 if the item is affected by DIF induced by covariate G, or the expected item response curves at time T1 and T2 if the item is affected by recalibration response shift (RS) over time

In case of absence of DIF/RS, the ICC between both groups/both time points completely overlap (same expected item response is obtained for the same level of anxiety or depression between groups or over time, see Fig. 3A). Whereas DIF or recalibration RS, operationalized by difference in item threshold parameters between group or over time, will result graphically in differences in the ICC (and the CCC), see Figs. 3B, C, D for an illustration. Hence, in case of DIF or RS, different expected item responses can be obtained for the same level of anxiety or depression between groups or over time.

Depending on how the item threshold parameters vary across groups and/or over time, several situations may arise with respect to the ICCs. For instance, if recalibration RS or DIF affects all the item thresholds in the same way (i.e. same direction and magnitude for the difference/change in the item threshold parameters), the ICC giving the expected item response as a function of the latent variable will be translated horizontally (i.e. an offset, see Fig. 3B). However, if the item thresholds are affected differently (varying direction and/or magnitude), the impact can go beyond simple translation—leading to changes in the slope or even the overall shape of the ICC (Figs. 3C and D, respectively).

#### Statistical tests involved in the ROSALI procedure to detect DIF and RS

As depicted in Fig. 1, both algorithms of ROSALI start with an overall test for the occurrence of DIF (algorithm #1) or recalibration RS (algorithm #2). These overall tests are performed by comparing two models through a likelihood-ratio test:

- **Algorithm #1, overall DIF occurrence?**
  - Fully non-invariant model = ‘cross-sectional model assuming that all items are affected by DIF'^1^
  - versus
  - Fully invariant model = ‘cross-sectional model assuming the absence of DIF’.
- **Algorithm #2, overall RS occurrence?**
  - Fully non-invariant model = ‘longitudinal model assuming that all items are affected by recalibration RS'^2^
  - versus
  - Fully invariant model = ‘longitudinal model assuming the absence of recalibration RS’.

If overall occurrence of DIF/recalibration RS is evidenced, ROSALI then performs an iterative step to determine which items show DIF/recalibration RS, during algorithm #1 and #2, respectively.

- **Iterative step of algorithm #1: which items show DIF?**
  Starting from a cross-sectional model assuming the absence of DIF (i.e. a model where all item thresholds $\:{\delta}_{jp}$ are assumed constant across G0 and G1), this iterative step relies on statistical testing to identify DIF items by estimating new models: one for each item under investigation for DIF, where its thresholds parameters are freely estimated across the two groups. For each of the new models, a statistical test is performed to determine whether the thresholds of the item under investigation vary significantly between G0 and G1 (the null hypothesis is H_0_: “For all $\:p$, item threshold parameters among G0 [$\:{\delta}_{j{p}_{0}}$] = item threshold parameters among G1 [$\:{\delta}_{j{p}_{1}}$]”, chi-squared distributed test statistic under the null hypothesis). At each iteration, DIF is assumed on the item associated with the most significant test after Bonferroni adjustment for multiple testing. The detection process continues and each iteration of this iterative step accounts for the DIF effects found during the previous iterations. The iterative step ends as soon as no significant tests are evidenced, or just after evidencing DIF on all but one item.

- **Iterative step of algorithm #2: which items show recalibration RS?**
  This iterative step operates as the one of DIF described above. It starts from a longitudinal model assuming no recalibration RS (but accounting for the DIF effects at time T1 found during algorithm #1), estimates new models: one for each item under investigation for recalibration RS (where its thresholds parameters are freely estimated over time), performs statistical testing to determine whether the thresholds of the item under investigation vary significantly over time, and assumes recalibration RS on the item associated with the most significant test after Bonferroni adjustment at each iteration. Of note, further statistical testing is performed to determine whether the recalibration detected occurs similarly in both groups or differently. The detection process continues and each iteration of this iterative step accounts for the RS effects found during the previous iterations. The iterative step ends as soon as no significant tests are evidenced, or after evidencing RS on all but one item.
  Further details on the ROSALI procedure can be found elsewhere [30, 31]. Of note, the application of the ROSALI procedure presumes that the PCM, fits adequately the data at T1. Hence, before conducting DIF and RS detection with the ROSALI procedure, we assessed the fit for both subscales of the HADS at T1 (one-month post-diagnosis) using fit indices based on residuals, namely inlier-sensitive INFIT and outlier-sensitive fit OUTFIT [48, 49]. As a result, item 11 “I feel restless as if I have to be on the move” was discarded from further analysis due to item misfit at T1 in both groups (large INFIT and OUTFIT, indicating that the item responses were more random than predicted by the model). Besides, two adjacent response categories were collapsed for some items to either deal with dysfunctioning items (i.e. items showing disordered thresholds, for instance $\:{\delta}_{j1}>\:{\delta}_{j2}$) or to ensure that all frequencies within the two-way contingency table “item response*cancer type” were above 0 at each time point. All collapsing performed are summarized in Table S1 (supplementary materials). Interested readers can find more details on PCM fit in [50, 51].

### Software

Statistical analyses were performed using Stata software version 17.0 (Stata Statistical Software: Release 17. College Station, TX: StataCorp LLC). To perform DIF and recalibration RS detection, we used the Stata module ROSALI, freely available on Boston College’s Statistical Software Components archive [52] and on the free pro-online website (https://pro-online.net/) for non-Stata users [53].

## Results

### Sample description

In total, 447 patients were included in the ELCCA cohort (337 breast cancer and 110 melanoma patients). Of note, one breast cancer patient did not provide any responses to the HADS at T1 and T2. This patient was therefore excluded from the analysis. The baseline characteristics of the 446 remaining patients appear in Table 1. Breast cancer patients were only female, while melanoma patients combined both male and female (62% and 38%, respectively). Overall, breast cancer patients were significantly more likely to have had children than melanoma patients.

**Table 1:** Baseline characteristics of patients according to cancer type (breast cancer or melanoma)

	Breast cancer *n* = 336	Melanoma *n* = 110	*P*-value
Age (years), mean (SD)	52.9 (8.3)	50.6 (12.4)	0.074
* N/A*	*1*	*0*	
Sex, *n* (%)			
Male	0 (0.0%)	68 (62.0%)	< 0.001
Female	336 (100.0%)	42 (38.0%)	
* N/A*	*0*	*0*	
Life as a couple, *n* (%)			
Yes	268 (81.2%)	88 (80.0%)	0.779
No	62 (18.8%)	22 (20.0%)	
* N/A*	*6*	*0*	
Children, *n* (%)			
Yes	299 (90.6%)	88 (80.0%)	0.003
No	31 (9.4%)	22 (20.0%)	
* N/A*	*6*	*0*	

*SD* standard deviation, *n* sample size/frequency, *%* percentage, *N/A* Not available

### DIF and RS detection in the anxiety subscale

**Fig. 4:**
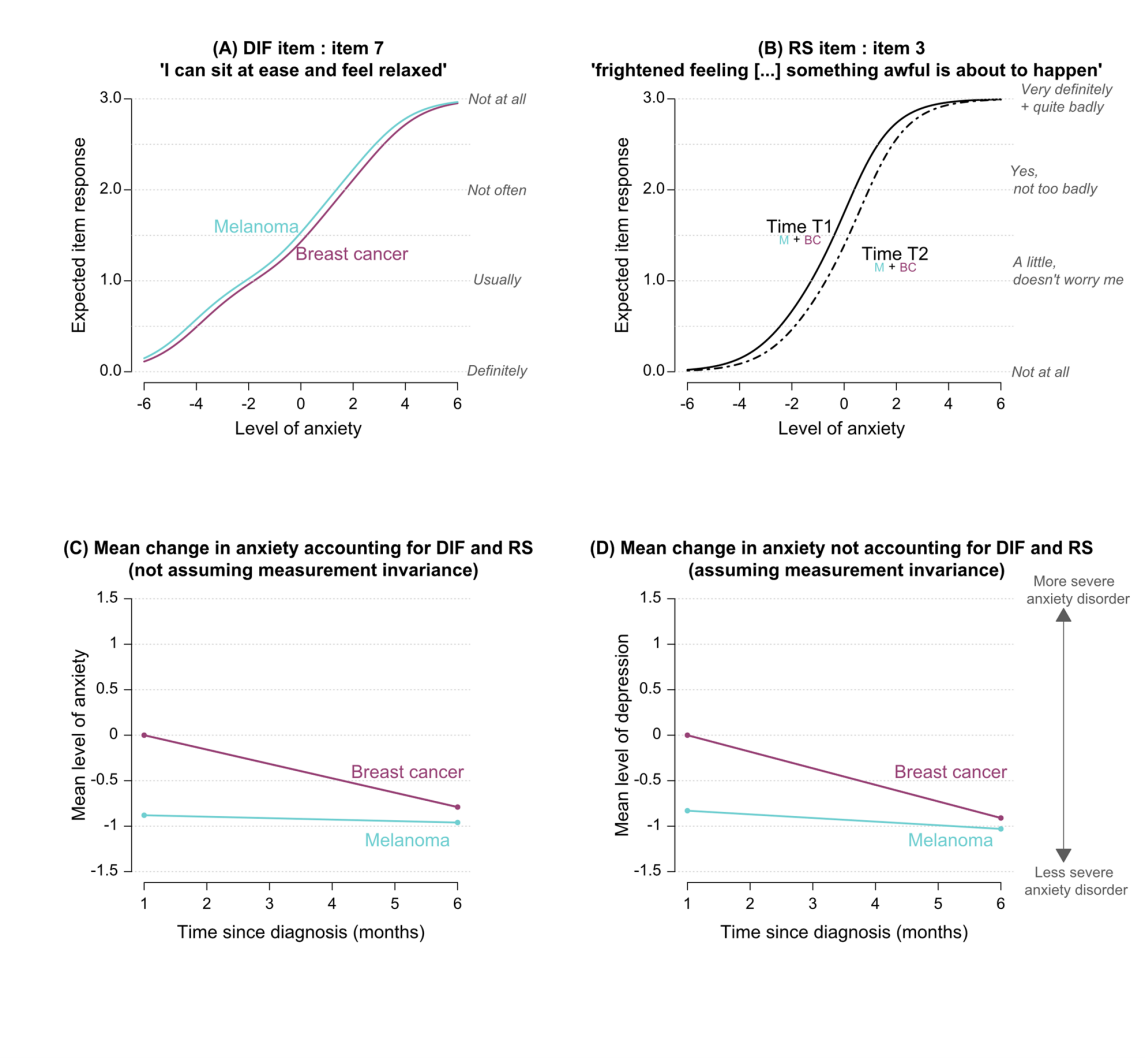
ROSALI procedure results for the subscale ‘*Anxiety*’ of the HADS. Graph (**A**): expected item response curve of item 7 “*I can sit at ease and feel relaxed*” in melanoma and breast cancer patients (item affected by differential item functioning (DIF) according to cancer type). Graph (**B**): expected item response curve of item 3 “*I get a sort of frightened feeling as if something awful is about to happen*” at time T1 and T2 in melanoma and breast cancer patients (item affected by recalibration response shift (RS) occuring similarly in both types of cancer). Graphs (**C**) and (**D**): mean change in the anxiety level among melanoma and breast cancer patients assuming (**D**) or not (**C**) measurement invariance. *Notes: M = Melanoma*,* BC = Breast cancer*,* DIF = Differential item functioning*,* RS = Response shift (recalibration)*,* T1 = 1 month after diagnosis*,* T2 = 6 months after diagnosis*

DIF between breast cancer and melanoma patients was found by ROSALI at T1 (i.e. one-month post-diagnosis) on item 7: “*I can sit at ease and feel relaxed*”. The item threshold parameters among melanoma patients at T1 (denoted $\:{\delta}_{7{p}_{\:M}\:}^{\left({T}_{1}\right)},\:$ M stands for melanoma) were significantly lower than among breast cancer patients (denoted $\:{\delta}_{7{p}_{\:BC}\:}^{\left({T}_{1}\right)}$, BC stands for breast cancer); for all $\:p$, $\:{\delta}_{7{p}_{\:M}\:}^{\left({T}_{1}\right)}-{\delta}_{7{p}_{\:BC}\:}^{\left({T}_{1}\right)}=\:-0.32,\:$standard error = 0.16. Hence, when patients had the same level of anxiety at T1 (e.g. an anxiety level equal to 0, see Fig. 4A), melanoma patients were more likely to choose a higher response category (i.e. reported to be less able to seat at ease and relax) than breast cancer patients. Of note, the same DIF remained on that item at T2 (i.e. 6-months post-diagnosis).

Similar recalibration RS was found in both groups on item 3: “*I get a […] frightened feeling as if something awful is about to* happen”. The item threshold parameters significantly increased between T1 and T2 (for all $\:p$ and in both groups, $\:{\delta}_{3p}^{\left({T}_{2}\right)}-{\delta}_{3p}^{\left({T}_{1}\right)}=\:+0.56,\:$standard error = 0.12). Hence, when patients had the same level of anxiety at T1 and T2 (e.g. an anxiety level equal to 0 at T1 and T2, see Fig. 4B), melanoma and breast cancer patients tended to give more optimistic answers at T2 than T1. No other recalibration RS effects were identified. Estimates of DIF and RS effects are available in the supplementary materials (Tables S2 and S3) alongside the item threshold parameter estimates.

Overall, DIF and recalibration RS effects did not significantly impact the comparisons between groups and over time, see Figs. 4C and D. Indeed, despite a moderate overestimation of the decrease in anxiety over time when wrongly assuming measurement invariance (i.e. assuming the absence of DIF and RS), the conclusions were similar, in terms of statistical significance, whether or not DIF and RS were taken into account (see Table 2).

**Table 2:** Estimates of group effect (melanoma versus breast cancer[reference]) and time effect (T2: 6-month versus T1: 1-month post-diagnosis[reference]) depending on whether or not differential item functioning (DIF) and response shift (RS) were taken into account

	Accounting for DIF and RS (i.e. not assuming measurement invariance)			Ignoring DIF and RS (i.e. assuming measurement invariance)		
	Estimate	SE	*P*-value	Estimate	SE	*P*-value
Anxiety						
Group effect (melanoma versus breast cancer)						
at T1	-0.88	0.17	< 0.001	-0.83	0.17	< 0.001
at T2	-0.16	0.19	0.398	-0.12	0.19	0.534
Time effect (T2 versus T1)						
among breast cancer	-0.79	0.08	< 0.001	-0.91	0.08	< 0.001
among melanoma	-0.08	0.14	0.575	-0.20	0.14	0.138
Depression						
Group effect (melanoma versus breast cancer)						
at T1	-0.12	0.18	0.511	-0.12	0.18	0.507
at T2	0.40	0.19	0.034	0.25	0.17	0.145
Time effect (T2 versus T1)						
among breast cancer	0.003	0.10	0.974	0.34	0.09	< 0.001
among melanoma	0.52	0.16	0.001	0.71	0.15	< 0.001

*T1* 1-month post-diagnosis, *T2* 6-month post-diagnosis, *DIF* differential item functioning, *RS* response shift, *Group effect* difference in the mean level of anxiety or depression between melanoma and breast cancer patients, *Time effect* change in the mean level of anxiety or depression between T1 and T2, Ignoring DIF and RS: group and time effects estimates obtained by assuming that item threshold parameters do not vary according to cancer type or measurement occasion (i.e., assuming measurement invariance), Accounting for DIF and RS: group and time effects estimates adjusted for DIF and RS effects found by the ROSALI procedure (i.e., not assuming measurement invariance)

Accounting for the detected DIF and RS, at T1, melanoma patients were, on average, significantly less anxious than breast cancer patients (-0.88, *p* < 0.001). The mean level of anxiety remained stable for melanoma patients between T1 and T2 (-0.08, *p* = 0.575) whereas it decreased for breast cancer patients (-0.79, *p* < 0.001), leading to similar mean anxiety levels between the two groups at T2 (-0.16, *p* = 0.398).

### DIF and RS detection in the depression subscale

**Fig. 5:**
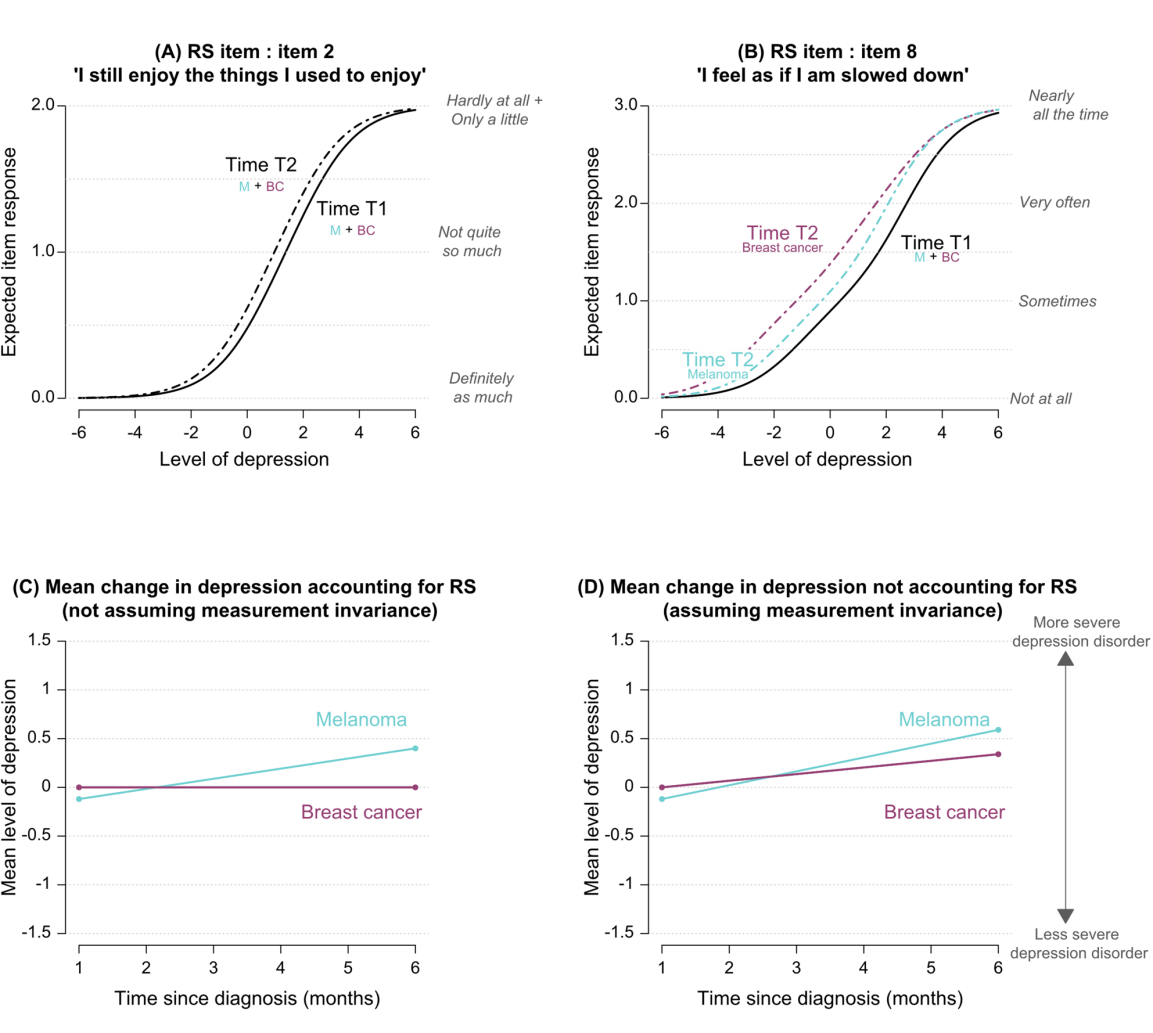
ROSALI procedure results for the subscale ‘*Depression*’ of the HADS. Graph (**A**): expected item response curve of item 2 “*I still enjoy the things I used to enjo*y” at time T1 and T2 in melanoma and breast cancer patients (item affected by recalibration response shift (RS) occuring similarly in both types of cancer). Graph (**B**): expected item response curve of item 8 *"I feel as if I am slowed down" *at time T1 and T2 in melanoma and breast cancer patients (item affected by recalibration response shift (RS) occuring differently in both types of cancer). Graphs (**C**) and (**D**): mean change in the depression level among melanoma and breast cancer patients assuming (**D**) or not (**C**) measurement invariance. *Notes: M = Melanoma*,* BC = Breast cancer*,* DIF = Differential item functioning*,* RS = Response shift (recalibration)*,* T1 = 1 month after diagnosis*,* T2 = 6 months after diagnosis*

No DIF was found for depression. Recalibration RS was detected on two items (see Figs. 5A, B). It occurred similarly in the two groups for item 2 “*I still enjoy the things I used to enjoy*” and differently for item 8 “*I feel as if I am slowed down*”. Regarding item 2 (“*I still enjoy the things I used to enjoy*”), its threshold parameters significantly decreased between T1 and T2 (for all $\:p$ and in both groups, $\:{\delta\:}_{2p}^{\left({T}_{2}\right)}-{\delta\:}_{2p}^{\left({T}_{1}\right)}=\:-0.41,\:$standard error = 0.14). Hence, when melanoma and breast cancer patients had the same level of depression over time (e.g. a depression level equal to 2 at T1 and T2, see Fig. 5A), they were more likely to report not being able to enjoy things as much as they used at T2 compared to T1. As for item 8 (“*I feel as if I am slowed down*”), its threshold parameters were also significantly decreased between T1 and T2, but to a larger extent among breast cancer patients than among melanoma patients:

- Melanoma patients: for all $\:p,$ $\:{\delta\:}_{8{p}_{\:M}\:}^{\left({T}_{2}\right)}-{\delta\:}_{8{p}_{\:M}\:}^{\left({T}_{1}\right)}=\:-0.66,\:$standard error = 0.21.
- Breast cancer patients: item threshold parameters were shifted between T1 and T2 (values ranging from − 1.59 to -0.57).

Therefore, breast cancer patients and, to a lesser extent, melanoma patients were more prone to report being slowed down at T2 as compared to T1, especially for low-to-mild levels of depression (i.e. level of depression below 2, see Fig. 5B). Estimates of all DIF and RS effects are available in the supplementary materials (Tables S4 and S5).

Recalibration RS effects had some impact on comparisons between groups and over time; see Figs. 5C and D, with different conclusions depending on whether RS was taken into account or not. Ignoring RS would have led to an overestimation of the increase in breast cancer patients’ mean depression level and would have masked the difference between groups at T2. Accounting for RS, changes in the mean levels of depression over time differed between groups. It remained stable among breast cancer patients (0.003, *p* = 0.974) while it increased in melanoma patients (0.52, *p* = 0.001), who were significantly more depressed than breast cancer patients at T2 (0.40, *p* = 0.034).

## Discussion

DIF and RS were identified in PROM measuring anxiety and depressive symptoms in breast cancer and melanoma patients between one- and six-month post-diagnosis. As DIF and RS had either a moderate or a significant impact on the results, it could have led to erroneous conclusions regarding time and group effects, if ignored. The ROSALI method based on RMT was used to detect and interpret DIF and recalibration RS, and to provide estimates of changes in anxiety and depression levels over time, accounting for DIF and recalibration RS, where appropriate.

### Detection and interpretation of DIF and RS – Clinical and methodological considerations

Melanoma patients reported being less able to feel relaxed^3^ than breast cancer patients at each time point (i.e. DIF) and both groups expressed feeling less frightened^4^ at T2 than T1 (i.e. RS), for a similar level of anxiety between groups and over time, respectively. Having difficulty to feel calm and relaxed, reflecting anxiety, was found to be prevalent in melanoma patients, especially before and during treatment, in a recent systematic review and meta-analysis [54]. Physical techniques, including relaxation training, as part of Cognitive Behavioral Therapy interventions could be beneficial for patients [55], particularly during vulnerable periods (e.g. diagnosis, treatment). Reporting being less frightened, as melanoma and breast cancer patients go through their treatment journey, may be indicative of the confidence they have in healthcare. It may also reflect a change in the meaning of the item wording over time (e.g. the “awful thing” may have already happened, i.e. cancer) or in response options where, e.g., “A little, but it doesn’t worry me” may not be interpreted in the same way at T2 and T1. Interestingly, the same kind of trend was previously found on part of this cohort regarding emotional functioning (EF) [31] where melanoma patients indicated being more irritable than breast cancer patients (i.e. DIF) and both groups expressed less worries at one-year as compared to one-month post-diagnosis (i.e. RS), when they had equivalent EF levels between groups and over time, respectively.

Concurrently, patients in both groups were more likely to indicate they didn’t appreciate the things they used to as much^5^ at T2 than T1, for a similar level of depression over time. Patients were also more inclined to report being slowed down^6^ at T2 compared to T1, especially at low-to-mild levels of depression. These RS may be linked to cancer-related fatigue which is well-documented as being prevalent during the treatment period [56]. Regarding ‘enjoying things’, it may also be that patients have adapted to their fatigue and developed new interests which could then be related to post-traumatic growth^7^ [57], already recognized in cancer patients [58, 59] and assumed to be associated with enhanced psychological adaptation [55]. The observation that RS is more marked at low-to-moderate levels of depression may be related to the fact that these patients may be more able to become aware of slowdown than patients with more severe levels of depression, as this symptom is part of their condition.

Very few studies relate to RS in anxiety or depressive symptoms in breast cancer or melanoma patients [60]. Most studies exploring measurement invariance (mainly RS) have primarily studied breast cancer patients and very few melanoma patients. Moreover, RS was mostly investigated in the context of HRQoL in which recalibration RS was often detected, as reported in two recent systematic reviews [60, 61]. This can be explained by the fact that the most widespread methodological approaches for RS analyses still pertain to design-based methods and mainly the then-test approach. The then-test is a method that allows detecting and ‘adjusting’ for RS. It is often applied at the dimension level (i.e. on dimension scores), using data collected at two measurement occasions (e.g., before and after the catalyst suspected to induce RS) [4]. However, this approach could not be used in our analysis as it had not been planned in advance for the ELCCA study. Specifically, it would have required asking patients to: (i) evaluate their level of the target construct, e.g. anxiety, at one and six months after the cancer diagnosis (pre-test and post-test assessments, respectively), (ii) retrospectively re-evaluate their level of the target construct as it was at the pre-test, six months after the cancer diagnosis (then-test assessment). This retrospective assessment was not collected within the ELCCA study. In addition, this approach relies on two strong assumptions: (i) that post-test and then-test share the same internal standards (i.e. similar cognitive process underlying patients’ responses), and (ii) that patients can accurately recall their pre-test state when completing the then-test (i.e. absence of recall bias). Both assumptions have been previously challenged in studies using qualitative methods in cancer patients (including breast cancer patients for one study) [62, 63] and using quantitative methods in diverse populations [64, 65]. Moreover, as with all statistical methods, it assumed that the majority of the sample shows RS in the same dimensions and direction [66].

Several statistical methods can be used to detect and account for recalibration RS using data already collected, including latent variable based methods such as SEM following Oort’s procedure [15] and the ROSALI procedure based on RMT [29, 31]. We preferred the ROSALI procedure, despite the widespread use of SEM, as it has been shown in a Monte Carlo simulation study that ROSALI outperformed SEM in detecting item-level recalibration with polytomous items [29]. Besides, within another simulation study, ROSALI considering one binary covariate (such as cancer type as in the ELCCA study) was shown to perform well with low rates of incorrect recalibration RS detection (when recalibration was not simulated) and moderate to high rates of correct detection of recalibration when recalibration was simulated (the detection performance varied according to degree of heterogeneity of item-level RS) [30]. Of note, both simulation studies involved a scenario similar to the HADS questionnaire (i.e., 7 items with 4 response categories). These simulation results, while informative and reassuring as to ROSALI’s performance, should nevertheless be regarded as optimistic. In fact, they correspond to simulations in which the entire sample is assumed to be affected by RS, which of course is unrealistic and most probably not the case in the patients included in the ELCCA study. However, as ROSALI satisfactorily prevents erroneous detection of RS (as was evidenced by the low false detection rates), we can be fairly confident about the results regarding the lack of measurement invariance detected in our study.

### Impact of DIF and RS on changes in anxiety and depression – Clinical and methodological considerations

DIF and RS could either slightly (for anxiety) or significantly (for depression) impact the results regarding the estimates of group and time effects. For instance, accounting for RS enabled to highlight the differences in the depression trajectories of melanoma and breast cancer patients and the significantly higher level of depression in melanoma compared to breast cancer patients at six-months post-diagnosis. These results would not have been revealed if RS had been ignored.

Accounting for DIF and RS in the analyses showed that mean levels of anxiety and depression differed according to cancer type and time since diagnosis. Close to diagnosis, breast cancer patients were more anxious than melanoma patients. During follow-up, anxiety remained stable in melanoma patients and decreased in breast cancer, becoming similar to that of melanoma at 6-month post-diagnosis. An opposite trend was observed for the change in depression over time, with a stable trajectory for breast cancer but not for melanoma patients, who were more depressed than breast cancer patients at 6-month post-diagnosis. Cancer type and treatment period have been shown to have an impact on psychological distress of patients [39, 41, 67]. For instance, Ranieri et al. [67] conducted a study including women with early-stage diagnosis of breast cancer or melanoma; more psychological distress was reported by breast cancer patients compared to melanoma, which was related to lower body image perception. The prevalence of depression was also found to differ between cancer types and to be most prevalent during acute phases, e.g. before and during treatments [43]. However, all these studies were cross-sectional and did not took into account lack of measurement invariance nor, obviously, its impact on the estimates of the prevalence of anxiety or depression.

Impact of a lack of measurement invariance, such as DIF or RS, has been assessed in some simulation studies and in empirical data using latent variable models [29, 30, 68–71]. All simulation studies, whether in a SEM or IRT/RMT framework, have shown that DIF or RS could have a substantial impact on the estimation of the difference between groups and change over time of the target construct. Within the RMT framework, which corresponds to the theory of measurement on which ROSALI is based, recommendations have been made for characterizing DIF with meaningful consequences [72]. In particular, it is stipulated that if significant DIF is identified, its effect-size should be provided as well as how DIF is accounted for in the analyses, as we did in our study. Although, as far as we know, there are no such recommendations for RS to date, the importance of providing effect sizes and not just p-values when doing any analysis is of paramount importance. This consideration is not new, and still applies in a very general way today, as evidenced by both older and more recent publications [73, 74].

### Strengths and limitations

The main strengths of our study include investigating DIF and RS in a sample combining melanoma and breast cancer patients followed longitudinally and providing likely unbiased estimates (due to ignoring DIF or RS) of anxious/depressive symptoms between groups and over time. We used ROSALI, a method validated in simulation studies, with good performance to detect item-level recalibration RS, minimize false DIF detection, and provide unbiased estimates [30, 31] of the construct of interest in conditions similar to the ELCCA study. Moreover, interpreting DIF and RS allowed getting more insight into (hidden) differences in anxiety and depression between groups and over time in cancer patients. We therefore not only investigated lack of measurement invariance through the unique lens of measurement bias but also as a meaningful phenomenon that could enhance healthcare management [9, 75].

Our study is not without limitations. First, we only examined recalibration RS as ROSALI is based on RMT that do not allow investigating other types of RS, namely reprioritization and reconceptualization RS. The latter are more often explored at the dimension level [24, 76, 77] which provides valuable insights into RS but at the risk of missing RS occurring at the item level.

Second, although we tried to minimize alternative explanations of DIF and RS findings, i.e. false positive [75], by paying attention to model fit before undertaking the analyses and promoting a multidisciplinary interpretation of our results, complementary qualitative research interviews would be needed to provide further exploration of the plausibility of DIF and RS. For instance, the change in threshold parameters for the item “I feel as if I am slowed down” could reflect recalibration RS in depression, but could also result from differential effects of cancer-related fatigue over time. It may indicate that this item captures a mixture of depression and fatigue symptoms rather than being a ‘pure’ depression indicator. More generally, changes in item threshold parameters over time can arise from genuine recalibration but also from time-varying confounding by related constructs. Distinguishing between these interpretations requires integrating psychometric findings with clinical knowledge and, when possible, multidimensional modeling or auxiliary measures of potentially confounding constructs such as fatigue.

Third, as there were only women in the breast cancer group, gender bias cannot be ruled out; hence, the DIF and RS found may not only be related to cancer type but also to gender. Fourth, we chose to focus on the “before-during treatments” period and one may find it restrictive and wonder whether DIF/RS would also be found in the longer-term. While this can be seen as a limitation, it may also be seen as a strength. Indeed, RS is assumed to be triggered by a catalyst [4, 5, 75] which can be well-identified in our study as the beginning of treatments, making alternative explanation for RS less likely. Fifth, in line with the previous limitation, ROSALI can only be applied to date using two time points. Longitudinal RMT models have been extended for categorical outcomes, in continuous time, while accounting for the possible lack of measurement invariance, using the Generalized Structural Equation Modelling framework from Stata software (currently submitted, preprint in [78]). However, it has not been evaluated using simulation studies yet, which may be computer intensive. Finally, although the ELCCA study focuses mainly on patients’ experiences according to cancer type, other covariates could be worth investigating. For example, it may be that having children, which differed significantly between breast cancer and melanoma patients, could be associated with different patients’ perceptions of the items measuring anxiety and depression between groups (i.e. DIF) or over time (i.e. RS). The first part of the ROSALI procedure (i.e. DIF) has been extended to include two possibly correlated binary covariates, which effects can be simultaneously evaluated. Its performance were assessed in a simulation study [79] which showed that this extension prevents false detection of DIF (i.e. low false detection rates). However, it was noted that accurate detection of DIF requires quite large samples of over 400 individuals.

## Conclusion

The ROSALI procedure based on Rasch models (freely available at https://pro-online.net/), was used to detect, interpret, and take into account DIF and RS, where appropriate. DIF and RS were found in self-reports of anxiety and depression in melanoma and breast cancer patients, possibly revealing psychological distress but also psychological adaptation to challenging health events. DIF and RS can be meaningful and are important to consider to better understand patients’ experiences; they are still too often ignored which can lead to biased estimation of the target construct of interest and subsequent suboptimal healthcare decision-making [6, 7].

## Supplementary Information

Supplementary Material 1.

## Data Availability

No additional data is publicly available, but interested individuals can contact the authors with reasonable data requests. The ROSALI procedure based on Rasch models can be freely performed from https://pro-online.net/.

## Footnotes

1 In this model, we have to assume that there is no group effect on the latent variable level (identifiability constraint: μ_1_=0).

2 In this model, we have to assume that there is no time effect on the latent variable level in both groups.

3 Item wording: *I can sit at ease and feel relaxed*, labeling of response options: *0 = Definitely*,* 1 = Usually*,* 2 = Not often*,* 3 = Not at all*.

4 Item wording: *I get a sort of frightened feeling as if something awful is about to happen*, labeling of response options: *0 = Not at all*,* 1 = A little*,* but it doesn’t worry me*,* 2 = Yes*,* not too badly*,* 3 = Very definitely and quite badly.*

5 Item wording: *I still enjoy the things I used to enjoy*, labeling of response options: 0 = *Definitely as much*,* 1 = Not quite so much*,* 2 = Only a little + Hardly at all* (collapsed response categories).

6 Item wording: *I feel as if I am slowed down*, labeling of response options: *0 = Not at all*,* 1 = Sometimes*,* 2 = Very often*,* 3 = Nearly all the time*.

7 “…the experience of positive change that occurs as a result of the struggle with highly challenging life crises.” (26).
