# Supplementary material for: Rasch models to assess the impact of lack of measurement invariance and reveal hidden differences in anxiety and depression between groups and over time in patients with early-stage melanoma or breast cancer using the RespOnse Shift ALgorithm at the Item level (ROSALI)
Source: BMC Med Res Methodol. 2026 Jan 22;26:52. doi: 10.1186/s12874-025-02756-2 (PMC12961859; doi:10.1186/s12874-025-02756-2)
Supplement: Supplementary file 1 — Supplementary Material 1. [file 12874_2025_2756_MOESM1_ESM.docx]

Title: Rasch models to assess the impact of lack of measurement invariance and reveal hidden differences in anxiety and depression between groups and over time in patients with early-stage melanoma or breast cancer using the RespOnse Shift ALgorithm at the Item level (ROSALI)

-

Electronic Supplementary Materials

Investigation of the lack of measurement invariance among the items of the Hospital Anxiety and Depression Scale (HADS) in breast cancer and melanoma patients with the 'RespOnse Shift Algorithm at the Item level' based on the Rasch Measurement Theory (= ROSALI procedure)

## Group and time effects (on a given latent variable) involved in the longitudinal partial credit models estimated during the ROSALI procedure

During the ROSALI procedure, considering one binary covariate G (forming two groups G0 and G1) and two time points T1 and T2, longitudinal partial credit models are estimated to detect recalibration response shift over time (see equations #4 and #5 in the manuscript).

**From these models, different group and time effects can be derived:**

- $\boldsymbol{\beta}\mathbf{=}\boldsymbol{\mu}_{\mathbf{1}}^{\mathbf{(}\boldsymbol{T}_{\mathbf{1}}\mathbf{)}}\mathbf{-}\boldsymbol{\mu}_{\mathbf{0}}^{\mathbf{(}\boldsymbol{T}_{\mathbf{1}}\mathbf{)}}$ **:** the group effect at time T1, that is the difference in the mean level of the latent variable (e.g., anxiety/depression) between the group G1 and G0 at time T1.
- $\boldsymbol{\beta}\mathbf{+}\boldsymbol{\beta}_{\boldsymbol{inter}}\mathbf{=} \boldsymbol{\mu}_{\mathbf{1}}^{\mathbf{(T2)}}\mathbf{-}\boldsymbol{\mu}_{\mathbf{0}}^{\mathbf{(}\boldsymbol{T}_{\mathbf{2}}\mathbf{)}}$ **:** the group effect at time T2, that is the difference in the mean level of the latent variable (e.g., anxiety/depression) between the group G1 and G0 at time T2.
- $\boldsymbol{\mu}_{\mathbf{0}}^{\mathbf{(}\boldsymbol{T}_{\mathbf{2}}\mathbf{)}}\mathbf{-}\boldsymbol{\mu}_{\mathbf{0}}^{\mathbf{(}\boldsymbol{T}_{\mathbf{1}}\mathbf{)}}$ **:** the time effect among G0, that is the change over time in the mean level of the latent variable (e.g., anxiety/depression) among G0.
- $\boldsymbol{\mu}_{\mathbf{1}}^{\mathbf{(}\boldsymbol{T}_{\mathbf{2}}\mathbf{)}}\mathbf{-}\boldsymbol{\mu}_{\mathbf{1}}^{\mathbf{(}\boldsymbol{T}_{\mathbf{1}}\mathbf{)}}$**:** the time effect among G1, that is the change over time in the mean level of the latent variable (e.g., anxiety/depression) among G1. Of note, the time effect among G1 can be expressed by combining the time effect among G0 and $\beta_{inter}$: $\mu_{1}^{\left( T_{2} \right)}-\mu_{1}^{\left( T_{1} \right)}= \mu_{0}^{\left( T_{2} \right)}-\mu_{0}^{\left( T_{1} \right)}+ \beta_{inter}$.

**Hence, the interaction parameter** $\boldsymbol{\beta}_{\boldsymbol{inter}}\boldsymbol{\neq0}$ **accounts for the possibility that:**

- The group effect at time T2 may differ from the one at time T1
- The time effect between T1 and T2 varies between G0 and G1

A graphical representation of the group effects at T1 and T2 and the time effects among G0 and G1 is given in Figure S1.

**Figure S1:** Graphical representation of the group and time effects (on a given latent variable) involved in the longitudinal partial credit models estimated during the algorithm #2 of the ROSALI procedure which considers one binary covariate G (forming two groups G0 and G1) and to time points T1 and T2.

**
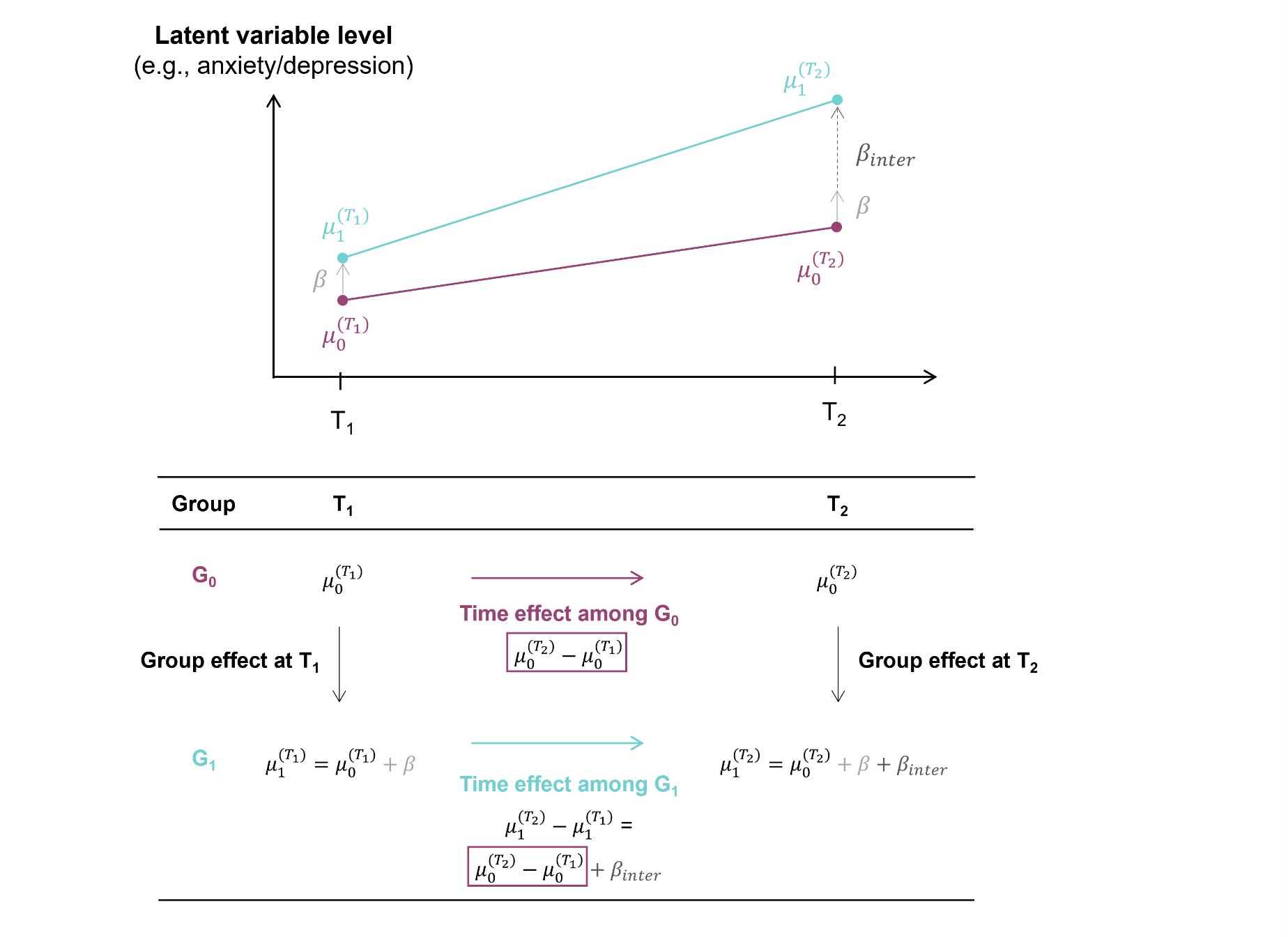
**

Time effect among G_1_

Time effect among G_0_

**Figure notes:** Constraint for identifiability is $\mu_{0}^{(T_{1})}=0$

## Preliminary analysis to perform before using ROSALI

Before conducting DIF and RS detection with the ROSALI algorithm, we assessed the fit to a Rasch model for both subscales of the HADS at T_1_ (one-month post-diagnosis) using fit indices based on residuals, namely inlier-sensitive INFIT and outlier-sensitive fit OUTFIT. As a result, item 11 *"I feel restless as if I have to be on the move"* was discarded from further analysis due to item misfit at T_1_ in both groups (large INFIT and OUTFIT, indicating that the item responses were more random than predicted by the model). Besides, two adjacent response categories were collapsed for some items to either deal with dysfunctioning items (i.e., items showing disordered thresholds, for instance $\delta_{j1}> \delta_{j2}$) or to ensure that all frequencies within the two-way contingency table "item response*cancer type" were above 0 at each time point. All collapsing performed are summarized in Table S.1.

**Table S.1:** Summary of the response categories which were collapsed

|  | **Before collapsing** | |  | **After collapsing** | |
| --- | --- | --- | --- | --- | --- |
|  | Code | Response categories |  | Code | Response categories |
| **(a) HADS-Anxiety** |  |  |  |  |  |
| 9. I get a sort of frightened feeling like butterflies in the stomach | 0  1  2  3 | Not at all  Occasionally  **Quite often**  **Very often** |  | 0  1  2 | Not at all  Occasionally  **Quite often + Very often** |
| 13. I get sudden feelings of panic | 0  1  2  3 | Not at all  Not very often  **Quite often**  **Very often** |  | 0  1  2 | Not at all  Not very often  **Quite often + Very often** |
| **(b) HADS-Depression** |  |  |  |  |  |
| 2. I still enjoy the things I used to enjoy | 0  1  2  3 | Definitely as much  Not quite so much  **Only a little**  **Hardly at all** |  | 0  1  2 | Definitely as much  Not quite so much  **Only a little + Hardly at all** |
| 4. I can laugh and see the sunny side of things | 0  1  2  3 | As much as I always could  Not quite so much now  **Definitely not so much now**  **Not at all** |  | 0  1  2 | As much as I always could  Not quite so much now  **Definitely not so much now +**  **Not at all** |
| 6. I feel cheerful | 0  1  2  3 | Most of the time  Sometimes  **Not often**  **Not at all** |  | 0  1  2 | Most of the time  Sometimes  **Not often + Not at all** |
| 10. I have lost interest in my appearance | 0  1  2  3 | I take just as much care as ever  **I may not take quite as much care**  **I don’t take as much care as I should**  Definitely |  | 0  1  2 | I take just as much care as ever  **I may not take quite as much care +**  **I don’t take as much care as I should**  Definitely |
| 12. I look forward with enjoyment to things | 0  1  2  3 | As much as I ever did  Rather less than I used  **Definitely less than I used**  **Hardly at all** |  | 0  1  2 | As much as I ever did  Rather less than I used  **Definitely less than I used + Hardly at all** |
| 14. I can enjoy good book or radio or TV program | 0  1  2  3 | Often  Sometimes  **Not often**  **Very seldom** |  | 0  1  2 | Often  Sometimes  **Not often + Very seldom** |

## Subscale Anxiety (HADS-A): size of the DIF and recalibration RS effects found

- **Item 7: affected by DIF according to cancer type**

Item wording: *I can sit at ease and feel relaxed*

Response categories: *0 = Definitely, 1 = Usually, 2 = Not often, 3 = Not at all*

The item has 3 thresholds: $\delta_{71}$, $\delta_{72}$ and $\delta_{73}$. The threshold estimates among breast cancer and melanoma patients at both time points are given in Table S.2 alongside the DIF size estimates.

**Table S.2**: Estimates of the item 7 (“*I can sit at ease and feel relaxed”*) thresholds among breast cancer (**BC**) and melanoma (**M**) patients at both time points alongside the estimates of the DIF size (DIF according to cancer type). The standard errors associated with these estimates are given in brackets. Of note, threshold estimates at **T_1_** and **T_2_** are identical as no recalibration response shift has been found between T_1_ and T_2_.

|  | **Thresholds** | | | | |  | **DIF size**  **at time T_1_ and T_2_** |
| --- | --- | --- | --- | --- | --- | --- | --- |
|  | **Time T_1_**  *Diagnosis + 1 month* | |  | **Time T_2_**  *Diagnosis + 6 months* | |  |  |
|  | **BC** | **M** |  | **BC** | **M** |  | (**M** versus **BC** *[reference]*) |
| $\delta_{71}$ | -3.93 (0.20) | -4.25 (0.23) |  | -3.93 (0.20) | -4.25 (0.23) |  | -0.32 (0.16) |
| $\delta_{72}$ | 0.35 (0.12) | 0.03 (0.18) |  | 0.35 (0.12) | 0.03 (0.18) |  | -0.32 (0.16) |
| $\delta_{73}$ | 3.01 (0.21) | 2.68 (0.25) |  | 3.01 (0.21) | 2.68 (0.25) |  | -0.32 (0.16) |

-0.32

-0.32

DIF:

- **Item 3: affected by recalibration RS between T_1_ and T_2_ occurring similarly among breast cancer and melanoma patients**

Item wording: *I get a sort of frightened feeling as if something awful is about to happen*

Response categories: *0 = Not at all, 1 = A little, but it doesn’t worry me, 2 = Yes, not too badly, 3 = Very definitely and quite badly*

The item has 3 thresholds: $\delta_{31}$, $\delta_{32}$, $\delta_{33}$. The threshold estimates at time T_1_ and T_2_ are given in Table S.3 alongside the recalibration RS size estimates.

**Table S.3**: Estimates of the item 3 (“*I get a sort of frightened feeling as if something awful is about to happen”*) thresholds among breast cancer (**BC**) and melanoma (**M**) patients at both time points alongside the estimates of the recalibration response shift (RS) size. The standard errors associated with these estimates are given in brackets. Of note, threshold estimates are equal between breast cancer and melanoma patients at both time points as no DIF according to cancer type has been found.

|  | **Thresholds** | | |  | **Recalibration RS size**  **among BC and M** |
| --- | --- | --- | --- | --- | --- |
|  | **Time T_1_**  *Diagnosis + 1 month* |  | **Time T_2_**  *Diagnosis + 6 months* |  |  |
|  | **BC** + **M** |  | **BC** + **M** |  | (**T_2_** versus **T_1_** *[reference]*) |
| $\delta_{31}$ | -2.19 (0.15) |  | -1.63 (0.15) |  | +0.56 (0.12) |
| $\delta_{32}$ | -0.09 (0.13) |  | 0.47 (0.15) |  | +0.56 (0.12) |
| $\delta_{33}$ | 0.72 (0.15) |  | 1.28 (0.17) |  | +0.56 (0.12) |

+0.56

Recalibration RS:

## Subscale Depression (HADS-D): size of the recalibration RS effects found

- **Item 2: affected by recalibration RS between T_1_ and T_2_ occurring similarly among breast cancer and melanoma patients**

Item wording: *I still enjoy the things I used to enjoy*

Response categories: *0 = Definitely as much, 1 = Not quite so much, 2 = Only a little + Hardly at all (collapsed response categories)*

The item has 2 thresholds: $\delta_{21}$ and $\delta_{22}$. The threshold estimates at time T_1_ and T_2_ are given in Table S.4 alongside the recalibration RS size estimates.

**Table S.4**: Estimates of the item 4 (“*I still enjoy the things I used to enjoy”*) thresholds among breast cancer (**BC**) and melanoma (**M**) patients at both time points alongside the estimates of the recalibration response shift (RS) size. The standard errors associated with these estimates are given in brackets. Of note, threshold estimates are equal between breast cancer and melanoma patients at both time points as no DIF according to cancer type has been found.

|  | **Thresholds** | | |  | **Recalibration RS size**  **among BC and M** |
| --- | --- | --- | --- | --- | --- |
|  | **Time T_1_**  *Diagnosis + 1 month* |  | **Time T_2_**  *Diagnosis + 6 months* |  |  |
|  | **BC** + **M** |  | **BC** + **M** |  | (T_2_ versus T_1_ *[reference]*) |
| $\delta_{41}$ | 0.31 (0.13) |  | -0.09 (0.15) |  | -0.41 (0.14) |
| $\delta_{42}$ | 2.44 (0.17) |  | 2.03 (0.17) |  | -0.41 (0.14) |

Recalibration RS:

-0.41

- **Item 8: affected by recalibration RS between T_1_ and T_2_ occurring differentially among breast cancer and melanoma patients**

Item wording: *I feel as if I am slowed down*

Response categories: *0 = Not at all, 1 = Sometimes, 2 = Very often, 3 = Nearly all the time*

The item has 3 thresholds: $\delta_{81}$, $\delta_{82}$and $\delta_{83}$. The threshold estimates at time T_1_ and T_2_ are given in Table S.5 alongside the recalibration RS size estimates.

**Table S.5**: Estimates of the item 8 (*I feel as if I am slowed down*) thresholds among breast cancer (**BC**) and melanoma (**M**) patients at both time points alongside the estimates of the recalibration response shift (RS) size. The standard errors associated with these estimates are given in brackets. Of note, threshold estimates are equal between breast cancer and melanoma patients at T_1_ as no DIF according to cancer type has been found at this time point. However, threshold estimates differ at T_2_ as the evidenced recalibration RS occurred differently between the two groups.

|  | **Thresholds** | | | |  | **Recalibration RS size**  **among BC** |  | **Recalibration RS size**  **among M** |
| --- | --- | --- | --- | --- | --- | --- | --- | --- |
|  | **Time T_1_**  *Diagnosis + 1 month* |  | **Time T_2_**  *Diagnosis + 6 months* | |  |  |  |  |
|  | **BC** + **M** |  | **BC** | **M** |  | (**T_2_** versus **T_1_** *[ref]*) |  | (**T_2_** versus **T_1_** *[ref]*) |
| $\delta_{81}$ | -1.23 (0.15) |  | -2.81 (0.26) | -1.89 (0.24) |  | -1.57 (0.28) |  | -0.66 (0.21) |
| $\delta_{82}$ | 2.03 (0.17) |  | 0.44 (0.18) | 1.37 (0.23) |  | -1.59 (0.21) |  | -0.66 (0.21) |
| $\delta_{83}$ | 3.39 (0.29) |  | 2.82 (0.27) | 2.74 (0.31) |  | -0.57 (0.38) |  | -0.66 (0.21) |

-1.57 ; -1.59 ; -0.57

-0.66

Recalibration RS:
